# The Tribology of Explanted Hip Resurfacings Following Early Fracture of the Femur

**DOI:** 10.3390/jfb6041021

**Published:** 2015-10-15

**Authors:** James K. Lord, David J. Langton, Antoni V.F. Nargol, R.M. Dominic Meek, Thomas J. Joyce

**Affiliations:** 1 Department of Biomedical Engineering and Mechanics, Virginia Tech, Blacksburg, VA 24061, USA; E-Mail: jklord@vt.edu; 2 North Tees Explant Centre (NTEC), Farndale House, University Hospital of North Tees, England TS19 8PE, UK; E-Mail: djlangton22@doctors.org.uk; 3 Joint Replacement Unit, University Hospital of North Tees, England TS19 8PE, UK; E-Mail: Antoni.Nargol@nth.nhs.uk; 4 Department of Orthopaedics & Trauma, Queen Elizabeth University Hospital, Glasgow G51 4TF, UK; E-Mail: rmdmeek@doctors.org.uk; 5 School of Mechanical and Systems Engineering, Newcastle University, England NE1 7RU, UK

**Keywords:** hip prosthesis, cobalt chromium, roughness, metal-on-metal, hip resurfacing, wear

## Abstract

A recognized issue related to metal-on-metal hip resurfacings is early fracture of the femur. Most theories regarding the cause of fracture relate to clinical factors but an engineering analysis of failed hip resurfacings has not previously been reported. The objective of this work was to determine the wear volumes and surface roughness values of a cohort of retrieved hip resurfacings which were removed due to early femoral fracture, infection and avascular necrosis (AVN). Nine resurfacing femoral heads were obtained following early fracture of the femur, a further five were retrieved due to infection and AVN. All fourteen were measured for volumetric wear using a co-ordinate measuring machine. Wear rates were then calculated and regions of the articulating surface were divided into “worn” and “unworn”. Roughness values in these regions were measured using a non-contacting profilometer. The mean time to fracture was 3.7 months compared with 44.4 months for retrieval due to infection and AVN. Average wear rates in the early fracture heads were 64 times greater than those in the infection and AVN retrievals. Given the high wear rates of the early fracture components, such wear may be linked to an increased risk of femoral neck fracture.

## 1. Introduction

Hip prostheses are an important tool for reducing pain and restoring function to patients with common musculoskeletal diseases such as arthritis. Recent advances have made hip replacement procedures increasingly common, with over 80,000 primary procedures in England, Wales and Northern Ireland in 2013 [1].

The reintroduction of metal-on-metal (MoM) hip prostheses has been shown to reduce volumetric wear compared with metal-on-polyethylene (MoP) implants [2,3]. Polyethylene wear has been linked to osteolysis [4] so the removal of the polyethylene bearing surface was intended to reduce this problem. The cobalt-chromium alloy used for femoral heads and acetabular cups in MoM hip prostheses can be polished to achieve an excellent surface finish and, when combined with highly controlled sphericity and clearance, allows for mild mixed or fluid film lubrication to be achieved during gait [2,5].

In the 1990s MoM hip resurfacing prostheses were introduced. These implants are designed to conserve bone and offer a more physiologically relevant load transfer [6], as well as to operate under fluid film lubrication for at least part of the gait cycle [7]. Such lubrication should reduce wear, compared with the boundary or mixed lubrication of conventional, smaller diameter total hip replacements [7]. However, maintaining fluid film lubrication in MoM hip resurfacings is dependent on a number of factors such as surface roughness, clearance between femoral head and acetabular cup, and material properties including Young’s modulus [8]. Hamrock and Dowson proposed an equation which has been used to calculate the lubrication regime in hip prostheses [9,10].

A number of different designs of MoM hip resurfacing prosthesis have been offered. The first, and still the market leader, is the Birmingham Hip Resurfacing (BHR) (Smith and Nephew, Warwick, United Kingdom) [11]. Two other common designs were: The Zimmer (Warsaw, IN, USA) Durom; and the DePuy (Leeds, UK) Articular Surface Replacement (ASR) [12]. It is important to note the engineering differences between these three designs, and these differences are summarised in Table 1. The Durom is made from wrought high carbon content (0.2%) cobalt-chromium-molybdenum alloy (CoCrMo) [6]. The BHR is made from cast CoCrMo while the ASR is also cast with heat treatment subsequently applied to the acetabular component [6]. The radial clearance, which has an influence on the theoretical lubrication regime, differs somewhat between the three designs (ASR ~50 μm, BHR ~100 μm, Durom ~75 μm) [6,8,13,14,15]. There are also differences in the arc of cover, which is the subtended angle of the articular surface. Depending on the size of the prosthesis, the ASR arc of cover can range from 148° to 160° [13]. BHR acetabular cups can range from 158° to 166° [13], while Duroms can also reach 166° [16]. These values are all significantly less than the 180° offered by traditional total hip arthroplasty acetabular cups [16]. In MoM hip resurfacings, decreased coverage arc has been associated with an increase in serum metal ion levels [17]. More recently it has been shown that elevated blood Cobalt concentrations were associated with an increased probability of early joint failure secondary to the development of an adverse local tissue response [18]. From clinical data, ASRs and Duroms have been noted for higher revision rates compared with the BHR. Data from the 2014 Australian Joint Registry showed that, at 7 years, revision rates were Durom 8.9%, ASR 23.9%, and BHR 5.0% [19]. The 2014 National Joint Registry (NJR) for England, Wales and Northern Ireland provided rates of 8.67%, 22.5% and 5.68% respectively at 7 years [1]. These revision rates are therefore likely linked to some of the engineering design differences.

**Table 1:** Summary of key design differences between the three models of hip resurfacing analyzed in this study.

Topic	ASR	BHR	Durom
Manufacture method	Cas-HIP ^1^/SA ^2^ cup	Cast	Wrought
Radial clearance (μm)	50	100	75
Arc of cover (°)	148–160	158–166	Up to 166
7 year revision rate:	–	–	–
AOA ^3^	23.9	5.1	9.0
NJR^4^	24.07	5.61	9.45

^1^ HIP = Hot Isostatic Pressurisation; ^2^ SA = Surface Annealing; ^3^ AOA = Australian Orthopaedic Association; ^4^ NJR = National Joint Registry for England, Wales and Northern Ireland.

Some short-term survival studies of MoM hip resurfacing prostheses have been encouraging [20,21]. Treacy *et al.* reported ten year survivorship for the BHR of 93.5%, using revision for any reason as the end point [22]. However, there are still many reported cases of failure among MoM hip resurfacing prostheses, with an average of 13.01% revision rate at ten years reported in England, Wales and Northern Ireland in 2014 [1]. A reason for some of the earliest of these failures is fracture of the femoral neck (*i.e.*, of the bone which locates the implant), a failure mode which is much more prevalent amongst resurfacing designs than conventional hip replacement [23]. Most fractures are termed “early”, occurring in the first few weeks [24,25] or months [26,27] after surgery, though they may still occur beyond a year. For example, Marker *et al.* reported an overall fracture risk of 2.5%, with half of these occurring in the first 12 months after surgery and the remainder later [28]. Several clinical studies have demonstrated the incidence of failure due to femoral neck fracture to be between 0.7% and 2.5% (Table 2) [21,24,25,26,27,28,29], and the 2010 Australian Orthopaedic Association arthroplasty register records the overall risk of fracture at 9 years as 2.6%, though the incidence increases rapidly in the first year after surgery and only very slightly thereafter [23]. The same source records cumulative revision rates for resurfacing devices in the same time frame as 7.2%. As such, fractures represent a significant percentage of overall resurfacing failures (35.6%). Several causes have been speculated for femoral neck fracture including surgical notching of the femoral neck and varus placement, both of which increase the stresses on the femur [26]. Risk factors identified include female gender [26,28], high Body Mass Index (BMI) [28] and surgeon learning curve [24,28], though the latter opinion has been disputed [26].

**Table 2:** Femoral neck fracture incidence from seven previous studies of hip resurfacings.

Lead Author	Year	Device	Number of Hips	Fracture Number (Incidence)	Mean Time to Fracture
Amstutz [24]	2004	Conserve Plus ^2^	400	3 (0.75%)	Not recorded
Treacy [21]	2005	BHR ^1^	144	1 (0.7%)	36 weeks
Cossey [25]	2005	BHR ^1^	407	7 (1.72%)	6 weeks
Shimmin [26]	2005	BHR ^1^	3497	50 (1.46%)	15.4 weeks
Marker [28]	2007	Conserve Plus ^2^	550	14 (2.55%)	16 weeks
Steffen [27]	2008	BHR ^1^	610	12 (1.97%)	Not recorded
Beaulé [29]	2004	Conserve Plus ^2^	119	1 (0.84%)	2 weeks

^1^ Birmingham Hip Resurfacing, Smith and Nephew, Warwick, United Kingdom; ^2^ Conserve Plus, Wright Medical, Arlington, TN, USA.

Given their recent introduction there are relatively few papers which have examined the tribology of *ex vivo* hip resurfacing prostheses [8,30,31]. Moreover, no papers have reported an examination of hip resurfacing prostheses removed due to early fracture of the femur. The aim of the study was to investigate if tribological data could offer insights into the causes of failure of these implants. The objectives were to measure the volumetric wear and surface roughness of *ex vivo* samples of three contemporary designs of hip resurfacing prostheses and compare results between four failure modes: early femoral neck fracture; adverse reactions to metal debris (ARMD) [32]; infection; and avascular necrosis (AVN). The roughness data was also used to determine the theoretical lubrication regime.

## 2. Results and Discussion

### 2.1. Wear Comparison

Descriptive statistics for each of the groups of fracture, AVN/infection, and ARMD femoral head are shown in Table 3. Duration *in vivo* was significantly smaller for the early fracture group (mean 3.7 months) than both AVN/infection (mean 44.4 months, *p* = 0.008) and ARMD (mean 30.9 months, *p* = 0.001). At 19.22 mm^3^, mean total wear volume of the ARMD retrievals was significantly greater than both AVN/infection (1.55 mm^3^, *p* = 0.001) and early fracture (6.53 mm^3^, *p* = 0.010). The difference in total wear volume between the early fracture group and the AVN/infection group was also significant (*p* = 0.018). When converted to wear rate, the early fracture group mean of 23.74 mm^3^/year was significantly greater than both AVN/infection (0.37 mm^3^/year, *p* = 0.005) and ARMD (8.29 mm^3^/year, *p* = 0.040). The difference in wear rates between ARMD retrievals and AVN/infection retrievals was also significant (*p* < 0.001). Patient metal ion levels at retrieval were greater in the ARMD group than AVN/infection for blood Cr (*p* = 0.001), blood Co (*p* = 0.007), serum Cr (*p* = 0.003) and serum Co (*p* = 0.007). The same was true between the ARMD and early fracture groups for ion levels (*p* = 0.001, *p* = 0.006, *p* = 0.002, *p* = 0.006, respectively). Comparison of the three failure modes found no significant differences in either unworn or worn λ (lambda ratio) values. The change in λ value between the unworn and worn regions was significant for the ARMD retrievals (*p* = 0.042) and the fracture retrievals (*p* = 0.028) but not for AVN/infection (*p* = 0.327). 

**Table 3:** Mean values for all hip resurfacings, grouped by failure mode.

Issue	Fracture	AVN/Infection	ARMD
Radius (mm)	24.31	21.87	23.28
Inclination (°)	43.8	45.6	51.0
Anteversion (°)	12.9	16.8	23.2
Duration (months)	3.7	44.4	30.9
Wear volume (mm^3^)	6.53	1.55	19.22
Wear rate (mm^3^/year)	23.74	0.37	8.29
Blood Cr (μg/L)	5.7	2.3	22.3
Blood Co (μg/L)	3.9	1.8	40.6
Serum Cr (μg/L)	5.7	2.9	29.2
Serum Co (μg/L)	4.2	2.3	38.2
Unworn λ	4.0	3.0	3.3
Worn λ	2.8	2.0	2.0

At a mean of 24.310 mm, the fracture retrievals had a larger radius than both ARMD (mean 23.280 mm) and AVN/infection (mean 21.869 mm), though the difference was not significant at the 95% confidence interval (*p* = 0.087 and 0.052, respectively). The ARMD retrievals were associated with cups implanted at significantly greater mean inclination angle (51.0°, *p* = 0.028) and anteversion angle (23.2°, *p* = 0.023) than the early fracture retrievals (43.8° and 12.9°, respectively). At 45.7°, the mean inclination angle of the AVN/infection group was not significantly different from the early fracture (*p* = 0.699) or ARMD (*p* = 0.277) group. The mean anteversion angle of 16.8° was significantly smaller than the ARMD group (23.2°, *p* = 0.029) but not significantly larger than the early fracture group (12.9°, *p* = 0.276). Implantation angles in the ARMD group were also significantly greater than the typical 45° inclination (*p* = 0.011) and 15° anteversion (*p* = 0.006) suggested as optimum [17,33,34].

*In vitro* studies suggest wear rates for MoM hip prostheses of 0.05–0.5 mm^3^/million cycles [33,34,35]. Hip resurfacing patients are generally younger and more active, and it has been suggested that for them 2.2 million cycles can be approximated to one year *in vivo* [36]. At a mean of 0.37 mm^3^/year, the AVN/infection group supports the results of simulator studies and the initial opinion that these failures were not device related. However, the ARMD and early fracture groups wore at significantly greater rates (8.29 and 23.74 mm^3^/year respectively or 23 and 64 times the rate shown by the AVN/infection group). These huge differences help to support the view that in the ARMD and early fracture groups, failure of the hip resurfacing was linked to metal wear from the articulating surface.

The detrimental effect of the steep inclination angle has recently been corroborated by a carefully controlled laboratory test [37]. Here, the difference in the steady-state wear of “correctly” positioned MoM hips (average 0.1 mm^3^/million cycles) and those at a high inclination angle (average 6.1 mm^3^/million cycles) was clear [37]. This 61-fold difference is of a similar order of magnitude to that for wear from the clinical explants reported here, namely the 64-fold difference between the AVN/infection group and the early fracture group of hip resurfacings.

Inclination and anteversion angles were high for the ARMD group. Acetabular cup angles of 45° and 15° are typically recommended [17,33,34] and the mean ARMD angles were 51.0° and 23.2°, respectively. It has been shown that these high angles are linked with high wear [13]. At 43.8° inclination and 12.9° anteversion, the early fracture group were implanted close to the recommended angles of 45° and 15°, respectively. Therefore wear in the early fracture group does not appear to be related to implantation angle.

There was a significant drop in λ value between the unworn and worn regions for both the early fracture (λ = 4.02 to λ = 2.77, *p* = 0.028) and ARMD (λ = 3.34 to λ = 2.03, *p* = 0.042) groups, although in both cases the shift was from fluid film to mixed lubrication during gait. The change in the AVN/infection group was not significant (λ = 3.00 to λ = 1.96, *p* = 0.416).

It could be claimed that the early fracture components were retrieved in the first few months after implantation and as such were in the so-called “running-in” phase where wear rates are temporarily increased before steadying at a lower level. This effect was demonstrated *in vitro* by Vassiliou *et al.*, who reported wear rates of 1.84 mm^3^ during the first million cycles of their BHR tests compared with 0.24 mm^3^ per million cycles over the final 2 million cycles of testing [38]. Heisel *et al.* demonstrated the same effect *in vitro* for five ASR prostheses, reporting an initial running-in wear of 1.42 mm^3^ over the first million cycles followed by steady state wear of 0.03 mm^3^/million cycles [36]. However, the truest test of all occurs when an artificial joint is implanted in human subjects. The 23.74 mm^3^/year wear rate of the early fracture retrievals is 13 times that reported in Vassiliou *et al.*’s *in vitro* study for the “high wear” running-in phase and 17 times that in Heisel *et al.*’s *in vitro* study. Therefore it is conceivable that such high wear rates *in vivo* may be a cause of prosthesis failure.

Might it be that the high wear is simply an effect of failure, rather than a cause? Might it be that, following fracture of the femur, the femoral head of the implant “rattled around” in the body and was damaged and worn. Evidence against this is twofold. Firstly, very little “rattling around” could occur after a patient’s femur has fractured. Secondly, if such “rattling around” damage did occur, leading to high wear, then why wasn’t high wear seen on the AVN retrievals which also suffered fracture of the femur?

Another counter point might be that these early fractures can be explained by clinical issues such as notching of the femoral neck, surgeon learning curve and other factors as outlined in the Introduction. While this may explain some fractures, if the explanted early fracture hips were caused by a surgical issue, then what explains the high roughness and wear seen? Why didn’t the early fracture hips show the low roughness and low wear seen on the AVN and infection retrievals?

In the wear analysis we undertake, we study not only the volume of material lost but also the wear scar morphologies; specifically the angle at which the maximum wear depths occur relative to the pole of the femoral head (though these values are not reported in this paper). We have never found any evidence to suggest that a displaced or undisplaced fracture leads to any difference at all in the distribution or magnitude of wear. Moreover, from the hip explants we have examined, we have no evidence to show that the position of the femoral head (either with a fixed stem shaft angle of a total hip replacement or the variable neck shaft angle of a hip resurfacing) affects edge wear at the acetabular cup.

### 2.2. Roughness Analysis

The average surface roughness values in the unworn and worn regions for each of the groups of early fracture, AVN/infection and ARMD femoral head components are shown in Table 4.

**Table 4:** Mean roughness values for each subset of components.

Failure mode	PV (μm)		RMS (μm)		Rsk	
	Unworn	Worn	Unworn	Worn	Unworn	Worn
Fracture	0.286	0.934	0.012	0.049	−2.225	−4.758
AVN/Infection	0.243	0.604	0.019	0.032	0.243	−2.869
ARMD	0.285	1.158	0.016	0.062	−1.075	−3.639

**Figure 1:**
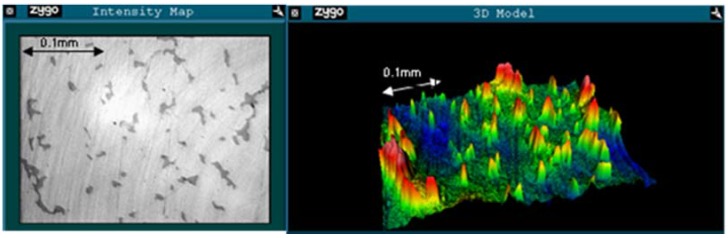
An example of a Zygo image of the unworn region of an ASR retrieved following femoral neck fracture after 2 months. *R*_a_ = 0.011 μm, RMS = 0.015 μm, PV = 0.114 μm, Rsk = 1.129. Intensity map shown to the left, 3D model to the right. Note that the surface is dominated by peaks (positive Rsk).

**Figure 2:**
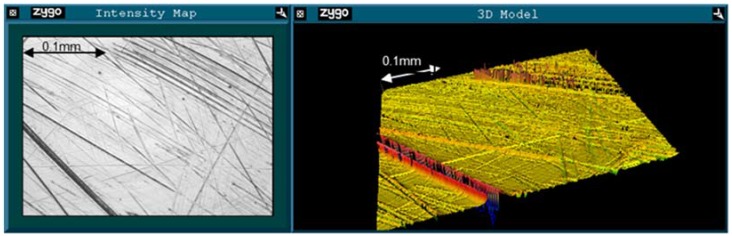
An example of a Zygo image of the worn region of the retrieval seen in Figure 1. *R*_a_ = 0.017 μm, RMS = 0.041 μm, PV = 1.145 μm, Rsk = −2.023. Note the lack of the protrusions seen in Figure 1 (and therefore negative Rsk).

Example images from the unworn and worn regions for an early fracture ASR are shown in Figure 1 and Figure 2, respectively. Surface roughness increased between the unworn and worn regions of the ARMD retrievals. Both RMS (0.016 μm to 0.062 μm, *p* = 0.015) and PV (0.285 μm to 1.158 μm, *p* < 0.001) increased, while Rsk decreased (−1.075 to −3.639, *p* = 0.005). In the early fracture group, only PV increased significantly (0.286 μm to 0.934 μm, *p* = 0.032), though RMS also increased (0.012 μm to 0.049 μm, *p* = 0.103) and Rsk decreased (−2.225 to −4.758, *p* = 0.185). The same patterns were seen in the AVN/infection group (RMS increased 0.019 μm to 0.032 μm, PV increased 0.243 μm to 0.604 μm, Rsk decreased 0.243 to −2.869). However, none of these changes were statistically significant at the 95% confidence interval (*p* = 0.127, *p* = 0.052, *p* = 0.096 respectively). In the unworn region, there were no significant differences in any measure of surface roughness between early fracture, AVN/infection or ARMD retrievals.

When all femoral head components were considered together, correlations in surface roughness were evident between the unworn and worn regions. This was true for RMS (0.720, *p* < 0.001) and PV (0.636, *p* < 0.001). RMS also correlated with PV in both the unworn (0.885, *p* < 0.001) and worn (0.891, *p* < 0.001) regions. Correlations were also evident between wear and surface roughness. Wear volume correlated with both RMS (0.611, *p* = 0.001) and PV (0.543, *p* = 0.003) in the worn region. Wear rate also correlated with RMS (0.469, *p* =0.012) in the worn region.

When compared to the unworn region, increased surface roughness was observed in the worn regions of all devices, regardless of failure mode. Roughening was most severe in the ARMD group (mean worn RMS = 0.062 μm), then the early fracture (mean worn RMS = 0.049 μm) and AVN/infection groups (mean worn RMS = 0.032 μm). This pattern follows the pattern of heaviest volumetric wear (19.22, 6.53 and 1.55 mm^3^, respectively). The early fracture retrievals showed higher roughness in the worn region than the AVN/infections, despite a significantly shorter duration *in vivo*. There was therefore a larger change in surface roughness over the short period of time prior to fracture. This data also means that roughening of surfaces *in vivo* was a relatively rapid process. This is in contrast to MoM hip simulator studies where, after an initial higher wear period, surfaces become smoother through a self-polishing action which leads to lower wear rates [5]. However the data reported here shows that 32 of 33 explanted femoral heads had roughened. In all but one case there was a drop in λ ratio in the worn region compared with the unworn. This single case may be evidence of the “self-polishing” phenomenon quantified recently on two MoM total hip prostheses [39]. Self-polishing was not seen on any of the other 32 components in this study.

Surface roughness was similar in the unworn region for all three failure modes (PV range: 0.243–0.286 μm, RMS range: 0.012–0.019 μm). Given that these areas were not wearing, the surface should be expected to be the same as that when first manufactured. As such, a similar surface roughness across all implants in the unworn area is to be expected regardless of failure mode and this indeed is what the measurements showed.

The increased roughness in the worn region of the early fracture retrievals suggests that even before the fracture of the femurs, the components were performing poorly. This would seem to be reflected in their increased wear volume (mean 6.55 mm^3^) compared with the AVN/infection retrievals (mean 1.55 mm^3^). A previous study investigating five ASR prostheses retrieved following pain and increased ion levels measured a mean femoral Ra of 0.063 μm (0.025 μm–0.135 μm) [8]. One case revised after 8 months (Cr = 35.9 μg/L, Co = 87.5 μg/L) (Ra 0.045 μm) also analysed the mating acetabular cup (*R*_a_ = 0.044 μm) and found a resulting λ ratio of 0.65. Although volumetric wear data was not available in that earlier paper, the authors postulated that such values of surface roughness and subsequent degradation of lubrication regime may have led to “greater than expected wear, concomitant higher ion levels in the patients, and may also be linked with early failure of these prostheses” [8]. The data reported in the current paper links increased surface roughness with high wear volumes. In the current study, wear volumes correlated with both RMS (0.479, *p* = 0.010) and PV (0.519, *p* = 0.005) in the worn region for all retrievals.

Across the 33 femoral heads reported on in this paper the λ ratios dropped from a mean of 3.60 in the unworn region (range 0.71–6.39) to 2.13 in the worn region (range 0.15–5.31). Although some elements of the Dowson-Hamrock equation for λ value were estimated due to the acetabular cups being unavailable, the shift in lubrication regime across many of the retrieved hip prostheses is of concern. Occurring *in vivo* this change may accelerate wear resulting in increased risk of early failure as well as the dangers of increased metal debris in the body.

Several previous studies have attempted to explain femoral neck fractures in hip resurfacing arthroplasty. Varus alignment and neck notching [26] during surgery were identified as risk factors, as were female gender and high BMI [28]. Poorly positioned components can also increase the stresses and strains on the femur, while high wear may lead to osteolysis even in metal-on-metal prostheses [40]. However, none of the previous studies have examined retrieved hip resurfacing prostheses for wear and surface roughness. Given the data contained in this study, it is suggested that high metal wear (64 times the volumetric wear rate that occurring with “normally functioning” resurfacings) may significantly increase the risk of early fracture of the femoral neck.

## 3. Experimental Section

### 3.1. Materials

Fourteen femoral components of hip resurfacing prostheses (six ASR, four BHR and four Durom) were obtained at revision surgery. The clinical data is shown in Table 5. In nine cases, the reason for revision was femoral neck fracture. The mean time to fracture was 3.7 months (range 2–7 months) and thus all fractures were said to be early. In three additional cases revision was due to AVN (38, 54 and 72 months). Avascular necrosis is a degeneration of the bone resulting from interrupted blood supply. As such, it is not an implant related failure mode and prostheses retrieved for AVN are not necessarily performing poorly. The remaining two prostheses were retrieved following diagnosis of an infection at the implantation site (28 and 30 months). As with AVN, retrieval following infection is usually not implant related. Given the relatively long duration *in vivo*, and the fact that the reason for revision was not considered to be related to the prostheses, the AVN and infection retrievals were grouped together for the purpose of analysis. In cases of femoral neck fracture and AVN it is usual to leave the original acetabular component in place and therefore only femoral components were available for study. Data from a previous study [41] which reported on nineteen ASR hip resurfacing femoral components retrieved after ARMD was used for comparison to the failure modes in the present study. Full ethical approval was obtained for the work undertaken.

**Table 5:** Clinical data for the 14 retrieved femoral components.

Diagnosis	Gender	Model	Duration (Months)	Inclination (°)	Anteversion (°)	Diameter (mm)
Fracture	Female	ASR	2	46.9	20.6	44.517
Fracture	Male	ASR	2	35.7	28.9	48.504
Fracture	Female	Durom	2	39.4	5.8	41.986
Fracture	Male	Durom	2	45.6	1.3	53.981
Fracture	Female	BHR	2.5	56.9	10.8	45.812
Fracture	Male	Durom	4	40.7	10.2	49.982
Fracture	Male	ASR	6	49.0	0.0	48.510
Fracture	Male	Durom	6	38.7	17.7	51.761
Fracture	Male	ASR	7	41.5	20.5	52.527
AVN	Male	ASR	38	38.0	17.0	46.499
AVN	Male	ASR	54	60.3	18.7	46.500
AVN	Female	BHR	72	40.2	18.0	37.841
Infection	Female	BHR	28	43.0	14.1	45.848
Infection	Female	BHR	30	46.7	16.0	42.006

### 3.2. Methods

#### 3.2.1. Clinical Data

Following initial implantation, cup inclination and anteversion angles were measured from X-rays using EBRA software [42]. At the time of revision surgery, blood metal ion levels (cobalt and chromium) were measured using a previously described method [43]. Duration *in vivo* was recorded.

#### 3.2.2. Wear Measurement

**Figure 3:**
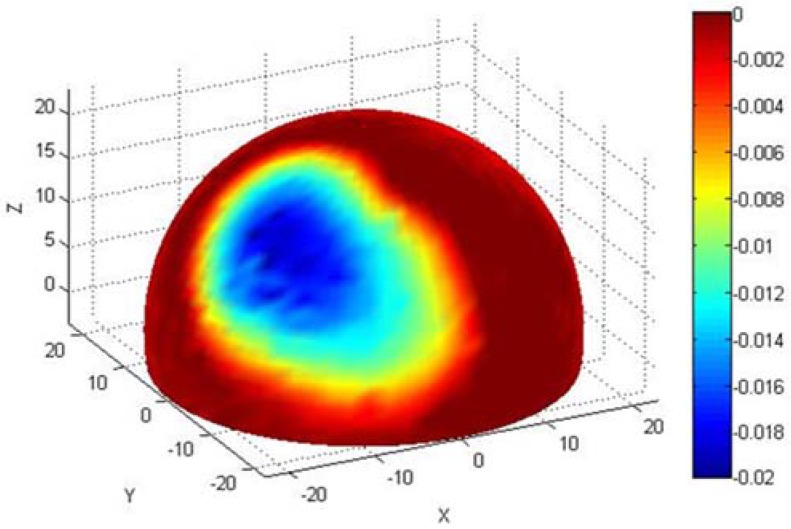
An example of the co-ordinate measuring machine (CMM) generated image for a BHR femoral head retrieved following femoral neck fracture after 2.5 months, showing linear wear depths (maximum depth = 20 μm, volumetric wear = 13.61 mm^3^) and allowing for classification of worn (green/blue) and unworn (red) regions.

After retrieval, all femoral heads were soaked in 10% formalin for one week before being rinsed thoroughly in water. Prior to measurement, the articulating surfaces were cleaned using acetone and a lint-free cloth in order to remove loose material and minimise spurious measurements. The femoral heads were then scanned using a Mitutoyo LEGEX322 co-ordinate measuring machine (CMM, Mitutoyo, Kawasaki, Japan) (scanning accuracy 0.8 μm) using a previously reported methodology [41]. This allowed calculation of total wear volumes and generated a model showing linear wear depths across the surface (Figure 3). This computer model allowed for the identification of worn and unworn regions on the surface.

#### 3.2.3. Roughness Measurement

The wear data was used to inform surface roughness measurements. These were taken using a Zygo New View 5000 non-contacting interferometer [8]. The articulating surface of each femoral head was again cleaned with acetone prior to measurements. Fifty readings were taken in total for each component and areas of interest were identified from the CMM analysis. Specifically, worn and unworn regions were defined for each head and then measurements taken within each of these regions. Each measurement contained approximately 76,000 data points in an area 0.317 mm by 0.238 mm from which surface roughness data was extracted. For each measurement, four different roughness parameters were recorded [44]:

- Peak to Valley (PV). The distance between the highest and lowest points on the surface. This gives the maximum size of defects in the scan area.
- Root Mean Square (RMS). The square root of the mean of the height differences squared. This gives a value for deviation in the surface height and accounts for both positive and negative variation (peaks and valleys).
- Skewness (Rsk). A measure of whether the surface is dominated by peaks (positive skew) or valleys (negative skew). A surface with negative skew is indicative of a series of valleys.
- Roughness average (Ra). The arithmetic average of the absolute height deviations.

*R*_a_ was used to calculate the lambda ratio (λ), using a modified version of the Hamrock-Dowson equation [8]. This equation allows for calculation of the minimum effective film thickness (*h*_min_) from:

(1) $$\frac{h_{\min}}{R_{x}}=2.80{\left(\frac{\eta u}{E*R_{x}}\right)}^{0.65}{\left(\frac{w}{R_{x}^{2}}\right)}^{-0.21}$$

Here, *R_x_* is the equivalent radius (m), η is the lubricant viscosity (Pa·s), u is the entraining velocity (m·s^−1^,), E* is the equivalent elastic modulus (Pa) and *w* is the load (N). Entraining velocity, *u*, varies with head diameter according to the formula:

(2) $$u=\frac{\omega d}{4}$$

Here, ω is angular velocity (rad/s) and d is head diameter (m). Equivalent elastic modulus, E*, depends on the material properties Young’s modulus, *E*, and Poisson’s ratio, *ν*:

(3) $$E*=\frac{E}{1-\nu^{2}}$$

The lambda ratio was then calculated from:

(4) $$\lambda =\frac{h_{\min}}{{\left[{\left(R_{a1}\right)}^{2}+{\left(R_{a2}\right)}^{2}\right]}^{0.5}}$$

where subscript 1 refers to the femoral head and subscript 2 refers to the acetabular cup. Some values had to be estimated for this calculation. Importantly, the cups were not available for analysis as they were not removed at the time of fracture of the femur. Therefore it was assumed that the *R*_a_ values on the cup were the same as the head. Similar *R*_a_ values of heads and cups after use have been reported from *in vitro* tests of MoM hip prostheses [45]. Additionally, clearance had to be estimated. Literature suggests a radial clearance of 50 μm for the ASR [6], 75 μm for the Durom [46] and 100 μm for the BHR [6,13]. Values for synovial fluid lubricant viscosity (0.0025 Pa·s), load (2500 N), Young’s modulus (210 GPa), Poisson’s ratio (0.3) and angular velocity (1.5 rad/s) were taken from the scientific literature [8,47]. It is however appreciated that values such as synovial fluid lubricant viscosity, loading and angular velocity will all vary between patients while loading and angular velocity will depend on activity.

### 3.3. Analyses

Differences between hip resurfacing designs subject to the three failure modes were analysed using two sample *t*-tests evaluated to the 95% confidence level (*p* = 0.05). Tests were conducted on duration *in vivo*, cup inclination and anteversion angles, head diameter, surface roughness, λ value, blood metal ion levels, wear volume and wear rate. Correlation tests were conducted across all designs to identify statistically significant factors affecting the wear rate of the implants. Quantitative roughness data was processed for the unworn and worn regions of each femoral head component. This allowed comparisons to be made between the worn and unworn regions of each component for each of the failure modes.

## 4. Conclusions

From the three groups of hip resurfacings measured (early fracture, ARMD, and AVN/infection) the early fracture group showed the highest wear rates. Comparative ratios were 64, 23 and 1. When surface roughness was considered, the early fracture group showed an increase in roughness in the worn area of the femoral head. This was despite their relatively short duration (mean 3.7 months) *in vivo*. Therefore it is suggested that a factor in early femoral neck fracture was the poor (high wear) performance of these hip resurfacings *in vivo*.
